# Similar Response Dynamics Represent Opposite Behaviors and Rewards in the Frontal Cortex

**DOI:** 10.1523/JNEUROSCI.1302-25.2026

**Published:** 2026-05-05

**Authors:** Pingbo Yin, Susanne Radtke-Schuller, Jonathan B. Fritz, Shihab A. Shamma

**Affiliations:** ^1^Institute for Systems Research & Department of Electrical and Computer Engineering, University of Maryland College Park, College Park, Maryland 20742; ^2^Department of Psychiatry & Center for Neurostimulation, University of North Carolina at Chapel Hill, Chapel Hill, North Carolina 27599; ^3^Center for Neural Science, New York University, New York 10012; ^4^Départment d’Étude Cognitives, L’École Normale Supérieure, PSL, Paris 75005, France

**Keywords:** auditory system, basal ganglia, frontal cortex, Go/NoGo task, negative reinforcement, positive reinforcement

## Abstract

The frontal cortex (FC) plays a pivotal role in adaptively controlling actions and their dynamics in response to incoming sensory signals. We explored FC encoding of identical stimuli and their behavioral consequences when they signified diametrically opposite responses depending on task context. Two groups of female ferrets performed Go-NoGo auditory categorization tasks with opposite contingencies and rewards and diverse stimuli. Remarkably, despite opposite stimulus-action associations, single-unit responses were similar across all tasks, being more sustained and stronger to Target sounds (signaling a change in action) than to Reference sounds (indicating maintenance of ongoing actions) especially during task engagement. Overall activity was composed of three distinct dynamic response profiles. Each corresponded to a separate neuronal cluster and exhibited a different role in relation to the succession of task events. Decoding based on the temporal structure of population responses revealed distinct decoders that were aligned to different task events. Similar to single-unit findings, the β-band power extracted from the FC local field potentials (LFPs) was strongly and similarly modulated during Target stimuli across all tasks despite opposite behavioral actions. In contrast, power in all other LFP frequency bands varied significantly across task stimuli and actions. Based on these findings, we propose the FC encodes a common, highly abstract representation of all the different behavioral tasks. We further outline a hypothetical model of pathway-specific functional projections from the tripartite FC neuronal clusters to the basal ganglia, consistent with previous evidence for the conjoint roles of the FC and striatum in adaptive motor control.

## Significance Statement

The frontal cortex (FC) encodes an abstract representation of perception and action with associated rewards and cognitive functions. Thus, even when ferrets perform opposite Go/NoGo behaviors, FC responses exhibit similar sequences of dynamic patterns from three cell clusters. The first component is phasic encoding stimulus category and the decision to maintain or change ongoing actions. The second is a rapid response suppression, initiated if the animal switches to a new action. The third is a buildup of excitatory activity as the animal sustains its new action. We propose a model for how such an abstract FC representation may emerge from separate functional projections from the FC clusters to the striatum, offering new insights into the FC role in behavioral control.

## Introduction

The frontal cortex (FC) has been implicated in many of the cognitive and executive control functions required for goal-directed behavior (Komura et al., 2001; Bruni et al., 2015; Duan et al., 2021; Friedman and Robbins, 2021), including decision-making (Coley et al., 2021; Liu et al., 2021), response inhibition (Schiller et al., 2014; Li et al., 2020), working memory (O’Reilly and Frank, 2006; Miller et al., 2018; Wilhelm et al., 2023), attentional control (Zikopoulos and Barbas, 2007; Gregorlou et al., 2014), and adaptive modulation of sensory filters (Banerjee et al., 2020). In the auditory system, cortical neurons can rapidly adapt their receptive field tuning and spectrotemporal selectivity reflecting changing stimulus context and task conditions (Fritz et al., 2003, 2005, 2007; David et al., 2012; Yin et al., 2014; Elgueda et al., 2019). This task-related receptive field plasticity may be shaped by changing functional connectivity between FC and auditory cortex (Fritz et al., 2010; Sheikhattar et al., 2018; Yin et al., 2020). This adaptive capacity is critical since context can transform the behavioral meaning of incoming stimuli and even cause the same sound to mean two opposite things in different circumstances.

In this study, we explored the role of the FC in this adaptive decision-making process by employing the same sounds to signify diametrically opposite meanings depending on task context and reward valence. In one behavioral paradigm, upon hearing a Target sound, animals initiated licking to obtain a water reward (positive reward; P-paradigm). In the other paradigm, animals learned to stop licking for water when presented with the same Target stimulus in order to avoid a mild shock (negative reward; N-paradigm). In an earlier study (David et al., 2012), we found that such different task reward structures and stimulus-action contingencies induced two strikingly distinct forms of receptive field plasticity in primary auditory cortex (A1). In light of the strong top-down projections from the FC to auditory cortex (AC) influencing dynamic sensory filters (Caras and Sanes, 2017; Bimbard et al., 2018; Schneider et al., 2018; Winkowski et al., 2018; Mittelstadt and Kanold, 2023; Macedo-Lima et al., 2024), we wondered whether the differential receptive plasticity was driven by distinct FC representations of the two opposite behavioral paradigms.

**Figure 1.**
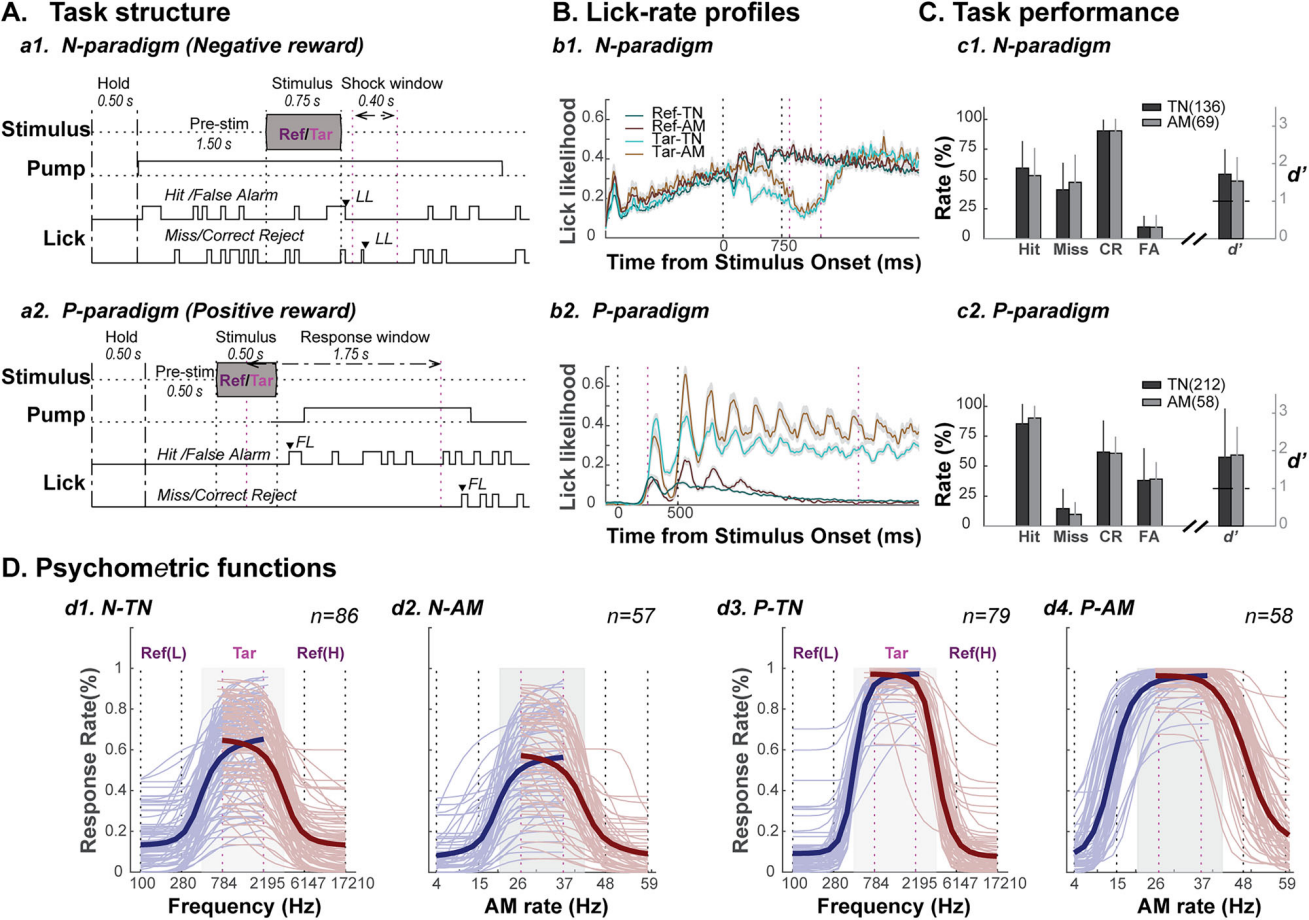
Behavioral performance on parallel auditory discrimination paradigms with Negative or Positive reward structures. ***A***, Task structure: four task variants are illustrated in this panel. In all, trials were initiated when the animal refrained from licking the waterspout for 0.5 s. In the **N-paradigm** (***a1***), after initial (0.5 s) lick withholding, the water pump was turned on, and animals began licking for water. A task sound (either **Reference** or **Target**) was presented 1.5 s later. Hence the status quo behavior in the N-paradigm was active licking prior to stimulus onset. Animals learned to stop licking after Target sounds, but to continue licking to **Reference** sounds. Ferrets scored a ***Hit*** and avoided a mild electric shock if they withheld licking throughout a brief post-Target stimulus shock window (0.4 s). However, if they licked during the shock window, they received a mild shock (***Miss***). In contrast, animals learned to continue licking after a **Reference** sound (***CR***, Correct Reject) and throughout the post-Reference stimulus period. If they stopped licking for the Reference, they received a 3–5 s timeout (***FA***, False Alarm) until the end of the trial. The last lick offset time (***LL***) before the end of the shock window was recorded and used to assess task performance. In contrast, in the **P-paradigm (*a2***), a **Reference** or a **Target** sound was presented 0.5 s after a trial was initiated (requiring initial lick withholding for 0.5 s). Hence the status quo behavior in the P-paradigm was no licking prior to stimulus onset. After a **Reference** stimulus, animals learned to continue withholding licking (***CR***) from the spout. If they licked after a **Reference** stimulus, they received a 3–5 s timeout (***FA***) by the end of the trial. After a **Target** sound, the animals learned to initiate licking within the response window (***Hit***), triggering the water pump to turn on for 1.2–1.8 s (providing ∼0.2 ml of water reward). The first lick onset time (***FL***) after a stimulus was recorded and used to assess task performance. ***B***, Lick-rate profiles: average licking profiles for all **Reference** and **Target** trials from all animals in each of the four task variants. The shaded area indicates standard errors. The vertical lines mark stimulus duration (dotted black) and response window (dashed magenta). ***C***, Task performance: the bar plots show the average response rate for four different trial outcomes (***Hit***, ***Miss***, ***CR***, and ***FA***) and task performance (*d′*) over all behavioral sessions from all different task variants. Performance of individual animals is shown in Supplementary Figure S2*A*. ***D***, Psychometric Functions (PFs): PFs computed by fitting the likelihood of behavioral response at the borders between **Reference** and **Target** stimulus categories for each behavioral session (thin lines), with the thick lines being the average **PFs** across all *n* sessions during physiological recordings. The light gray shaded area marks the borders between the **Target** category (intermediate region) and each of the low (blue lines) and high (red lines) **Reference** category regions. The vertical dashed lines indicate the tone frequencies [or amplitude-modulated (**AM**) noise rates] used during training. The plots display **PFs** from two animals, one for the N-paradigm (***d1*** and ***d2***) and another for the P-paradigm (***d3*** and ***d4***). Both animals were trained on the same task stimulus sets of tones (**TN**-tasks) and amplitude-modulated noise (**AM**-tasks). The individual **PFs** from the remaining animals are available in Supplementary Figure S2*B*.

Therefore, we trained two groups of ferrets on two opposite auditory categorical Go-NoGo paradigms, requiring each group to discriminate noncompact sound categories (Yin et al., 2016, 2020). Task stimuli varied along two acoustic feature dimensions: spectral frequency (TN-task) or temporal modulation rate (amplitude-modulated white noise, AM-task). As indicated above, in the P-paradigm group, ferrets learned to lick for water reward when Target stimuli were presented and refrained from licking to Reference stimuli. In contrast, the group that learned the N-paradigm performed the opposite behavior and refrained from licking for water when Target stimuli were presented but could lick freely to Reference sounds (Fig. 1*A*).

The most surprising finding was a remarkable similarity of single-unit response patterns in all behavioral contexts despite opposite auditory-motor associations in the two paradigms. This result suggests a highly abstract level of representation in the FC. Another interesting result was the discovery of three distinct neuronal clusters with temporally sequenced responses that aligned with the timing of behavioral responses of the animals in the two paradigms. A weighted composite of these three response clusters closely resembled β-band activity, derived from the accompanying local field potentials (LFPs) that have been shown to be a signature for either stopping ongoing actions or switching and initiating new actions (Picazio et al., 2014; Jahanshahi et al., 2015; Shin et al., 2017; Wessel and Aron, 2017; Horst et al., 2024; Polyakova et al., 2024).

To integrate all these findings, we propose a testable, hypothetical model in light of the role of the FC–basal ganglia (BG) loops (both cognitive and motor) in controlling and gating goal-directed behavior through three cortico-striatal pathways: the Hyperdirect, Direct, and Indirect pathways (Alexander et al., 1986; Alexander and Crutcher, 1990; Mink, 1996; Nambu et al., 2002, 2023; Nelson and Kreitzer, 2014; Baladron and Hamker, 2020; Hernandez-Martin et al., 2023; Redinbaugh and Saalmann, 2024). Many previous studies have explored the role of these descending cortico-striatal pathways in shaping behavior. According to classical Go-NoGo models, the Direct and Indirect pathways exert opposing control over movement (DeLong, 1990). However, more recent studies have shown that both pathways can be coactivated during intended movements (Cui et al., 2013), leading to cooperative, coactivation, or complementary encoding (Bariselli et al., 2019; Varin et al., 2023), or task-dependent models (Bolkan et al., 2022). Our experimental results in the FC and proposed model advance our understanding of the abstract FC representation and provide new insights into delineating the contributions of these cortico-striatal pathways to adaptive movement.

## Materials and Methods

All animal experimental procedures were conducted in accordance with the National Institutes of Health's Guide for the Care and Use of Laboratory Animals and were approved by the Institutional Animal Care and Use Committee (IACUC) of the University of Maryland.

### Animals

Five adult female ferrets (*Mustela putorius*) obtained from Marshall Farms were used in the current study. Ferrets were housed in pairs or trios in animal facilities accredited by the Association for Assessment and Accreditation of Laboratory Animal Care (AAALAC) and were maintained on a 12 h light/dark artificial light cycle. All ferrets began behavioral training between 1 and 2 years of age, with weights between 600 and 900 g. During initial behavioral training and the following behavioral physiological recording period, animals were placed on a weekly water control protocol, in which they received *ad libitum* water freely over weekends. On weekdays, they were brought to the laboratory for daily training/testing and returned to the animal facility after completion of their behavior session. While on training/testing during weekdays, the animals received water reward for correct trials during their behavior session. Additional water supplements were given after testing if an animal did not drink sufficiently during their daily behavior session. Animal health was carefully monitored daily by the experimentalists and the veterinary staff to avoid dehydration or weight loss (animals were maintained above 80% of *ad libitum* weight).

### Experimental apparatus

Behavioral training was conducted within a single-walled, sound-attenuated chamber (IAC). Ferrets were trained in a custom-built transparent Lucite testing box (18 cm width, 34 cm depth, 20 cm height), placed inside the booth. A lick-sensitive waterspout (2.5 cm × 3.7 cm) stood in front of the testing box, 12.5 cm above its base, and positioned so that animals could easily lick from the waterspout to obtain water through a small opening in the front wall of the box. The waterspout was connected to a computer-controlled water pump (MasterFlex L/S, Cole-Parmer) for one training paradigm (the Negative Reward or N-paradigm) or to a computer-controlled water dispenser (Crist Instrument) for the other training paradigm (the Positive Reward or P-paradigm). A custom-built interface box monitored the animal's lick behavior as measured by tongue contact with the waterspout and converted the licks to TTL digital signals which were fed back to the computer. A loudspeaker (Manger) was positioned 40 cm in front of the testing box for sound delivery during behavioral training, and the animal's behavior was monitored with a video camera, displayed graphically trial-by-trial, and continuously observed on a computer screen. All acoustic stimuli were generated using MATLAB (The MathWorks) at a 40 kHz sampling rate and ramped with 5 ms rise–fall times. Sounds were then digitized at 16 bit resolution through a NI-DAQ card, amplified, and delivered to the loudspeaker. All behavioral training and the following neurophysiological recordings were controlled and monitored through a custom-built MATLAB GUI. All related trial events were recorded and stored in the computer for further analyses.

### Behavioral paradigms

Two opposite variants of a Go-NoGo task with opposite stimulus-action behavioral contingencies were employed in the experiments in this study. Three ferrets (Nile, Bramble, Ganges) were trained on a three-range classification task using a positive-reinforcement paradigm (P-paradigm; for more details, see Yin et al., 2016). Animals learned to lick a waterspout (Go-behavior) upon hearing a “safe” Target sound to obtain a water reward (drops of 0.2–0.4 ml) and refrained from licking the waterspout (NoGo-behavior) following a “warning” Reference sound to avoid a 5–10 s timeout (Fig. 1*B*, top panel). Two other ferrets (Gong, Guava) were trained using a conditioned avoidance paradigm (N-paradigm; for more details, see Yin et al., 2020). In the N-paradigm, animals initiated licking the waterspout for water when the pump was turned on at trial onset, at a flow rate of 0.6–1.2 ml/min. Ferrets learned to stop licking (NoGo-behavior) after a “warning” Target sound to avoid a mild electric shock. In contrast, the presentation of a “safe” Reference sound signaled the animals to continue licking for water (Go-behavior). Failure to lick to the Reference stimulus led to a timeout after the trial (Fig. 1*A*, top panel). Hence, “Target” category sounds led to “Go-behavior” in the P-paradigm and “NoGo-behavior” in the N-paradigm. “Reference” category sounds in contrast led to “NoGo-behavior” in the P-paradigm and “Go-behavior” in the N-paradigm (Table 1). Therefore, in the two animal groups and associated behavioral paradigms, the same stimulus categories (Target or Reference) were associated with opposite behavioral actions (Go or NoGo licking responses).

**Table 1.** Training paradigms and stimulus sets

Ranges (category)	N-paradigm			P-paradigm			
	TN-task	AM-task	Behavior	TN-task		AM-task	Behavior
Low (Reference)			Go (licking)	****** 359 ******	****** 125 ******		NoGo (no licking)
				****** 402 ******	****** 176 ******	****** 4 ******	
	****** 100 ******	****** 4 ******		****** 450 ******	****** 249 ******		
	****** 280 ******	****** 15 ******		****** 504 ******	****** 350 ******	****** 15 ******	
				****** 565 ******	****** 494 ******		
				******633******	****** 697 ******		
Middle (Target)	****** 748 ******	****** 26 ******	NoGo (no licking)	****** 709 ******	****** 982 ******	****** 26 ******	Go (licking)
	****** 2,195 ******	****** 37 ******		****** 794 ******	****** 1,385 ******	****** 37 ******	
				****** 889 ******	****** 1,953 ******		
				****** 996 ******	****** 2,753 ******		
				****** 1,115 ******	****** 3,882 ******		
High (Reference)	****** 6,147 ******	******48******	Go (licking)	****** 1,249 ******	****** 5,474 ******	****** 48 ******	NoGo (no licking)
	****** 17,210 ******	****** 59 ******		****** 1,399 ******	****** 7,719 ******	****** 59 ******	
				****** 1,566 ******	****** 10,883 ******		
				****** 1,754 ******	****** 15,345 ******		
Animals	****** Guava Gong ******	****** Guava Gong ******		Nile Ganges	Ganges	Bramble	

In both training paradigms, the stimulus in each trial consisted of the presentation of a single sound (0.5 s duration in the P-paradigm and 0.75 s in the N-paradigm). Each trial in both paradigms was initiated when the animal refrained from licking the waterspout for at least 0.5 s. In the N-paradigm, trial initiation began with the water pump turning on, leading to animal licking before the presentation of the task sound. Thus the immediate prestimulus status quo behavior in the N-paradigm was licking. However, in the P-paradigm, a large drop of water was delivered only at the beginning of a task session; animals consumed the free water and then refrained from licking to initiate the first trial. Hence, the prestimulus status quo behavior in the P-paradigm was to refrain from licking.

The daily training session ended naturally when the animals had completed multiple trials (typically 100–200) and were no longer thirsty. The session ended when animals stopped engaging in the task and did not lick the waterspout for three consecutive trials despite the presence of a water stream in the N-paradigm or for three Target trials in the P-paradigm.

### Training procedures and stimulus sets

Animals were trained on two stimulus sets to categorize: pure tones or amplitude-modulated stimuli. One set consisted of pure tone stimuli that varied along the frequency dimension (TN-task), and the other consisted of amplitude-modulated noise stimuli that varied along the temporal dimension (AM-task). In the TN-task, the initial stimulus set in the N-paradigm consisted of six stimuli (Table 1) that spanned a 7-octave frequency range with 1.5 octave increments between the pure tone stimuli. The initial training sets were slightly different in the TN-task for the P-paradigm animals. Here the initial TN-task training set consisted of 15 pure tone stimuli with a varied frequency range. In one ferret (Ganges), the frequency range was 7 octaves as in N-paradigm training. For the other two ferrets (Nile and Bramble), the initial training set had a finer frequency resolution spanning only a 2.5-octave frequency range with 1/6 octave increments between adjacent stimuli (Table 1). The initial training set in the AM-task for both paradigms consisted of six stimuli ranging from 4 to 60 Hz amplitude modulation (100% depth) using white noise as carrier (Table 1). All stimulus sets were divided into three regions along the relevant Spectral or Temporal dimensions, where stimuli in the Middle region were labeled as “Target” stimuli and others in the two flanking regions (Low and High) were labeled as “Reference (non-Target)” stimuli (Table 1). As mentioned above, animals in the P-paradigm learned to lick the waterspout for water reward if the stimulus was in the “Target” category and to refrain from licking for the “Reference” category. In contrast, in the N-paradigm, animals learned to refrain from licking the waterspout for the Target stimulus category to avoid a mild shock and to lick freely for the Reference stimuli (Fig. 1).

The training procedures were as described in detail in our previous publications (Yin et al., 2016, 2020). Briefly, task training began after a 1–2 d habituation period, during which the animals became familiar with the testing environments (waterspout, speaker, light configuration, testing box dimensions, etc.) and were given positive reinforcement (liquid reward) in the behavioral training arena. For both behavioral paradigms, all ferrets were first trained on the three-range categorization TN-task with tone frequencies listed in Table 1. After reaching behavioral criterion, three animals (see Table 1 for details) were further trained on the AM-task using a set of six amplitude-modulated noise stimuli and learned to perform the three-range AM-task categorization. Intensity cues (initially 40 dB louder Target over Reference sounds) were used to direct the animal to attend the contrast between the sound categories at the earliest stage of the TN-task training. These intensity cues were gradually reduced and eventually completely removed over the course of training. No intensity cues were used for the AM-task training since all animals by that point in training had already learned the basic task structure following prior TN-task training. All five ferrets successfully learned both AM and TN categorization tasks to behavioral criterion (see below) and were able to generalize their performance to novel stimuli along the trained dimensions (some of these behavioral data have been previously published in Yin et al., 2016).

### Behavioral assessment and psychometric functions fitting

#### Behavioral assessment

Behavioral performance of animals performing the Go-NoGo tasks was assessed based on analysis of their stored digitized lick signals (the TTL signals) to task stimuli during daily task sessions. The timing of the rising edge of the first lick (***FL***) after each sound presentation in the P-paradigm, or the falling edge of the last lick (***LL***) before the end of the shock window in the N-paradigm, was recorded as the behavioral response in a trial (as illustrated in Fig. 1*A*,*B*), or in the absence of a lick, as no-response. A response that fell within a time window (0.25–2.0 s after sound onset) in the P-paradigm or before the shock window (0.85–1.25 s from sound onset) in the N-paradigm was defined as a Hit if the sound was a Target or a False Alarm if the sound was a Reference. Otherwise, if the response occurred after the time window in the P-paradigm or occurred within the shock window in the N-paradigm, it was defined as a miss after a Target or a correct reject after a Reference. The hit rate (HR), false alarm rate (FAR), miss rate (MR), and correct reject rate (CR) were computed for each behavioral session. Two metrics used for assessing animal behavioral performance were derived in relation to HR or FAR. One was the discrimination rate (DR) = HR * (1 − FAR) * 100% (Heffner and Heffner, 1995; Fritz et al., 2003). The other measure used was the *d′* = Φ^−1^ (HR) − Φ^−1^ (FR), where Φ^−1^ denotes the inverse cdf (cumulative distribution function) of the standard normal distribution. Proficient task performance on a behavioral session was defined as either or both DR > =40% or *d′* > = 1. The criterion for task mastery was the achievement of proficient performance in three consecutive training sessions, with >100 trials in each session.

#### Psychometric functions and fitting

The psychometric function (PF) describes the probability of the animal making a response as a function of the quantitative characteristic along the task-relevant stimulus feature dimensions (frequency in TN-task or amplitude-modulation rate in AM-task). We used a generic 4-parameter Logistic (sigmoid) ascending function to fit the lower boundary of the PF between low to middle range (function 1A) and the descending function for the upper boundary from middle to high range (function 1B):

$$R(x;\lambda ,\gamma ,\alpha ,\beta )=\gamma +(1-\lambda -\gamma )/(1+(\alpha /x{)}^{\beta }),\;(1A)$$

$$R(x;\lambda ,\gamma ,\alpha ,\beta )=(1-\gamma )-(1-\lambda -\gamma )/(1+(\alpha /x{)}^{\beta }).\;(1B)$$

In these two formulas, the parameter γ corresponds to the baseline rate or guess rate, reflecting the animal making a correct response purely by guessing irrespective of the stimuli. The parameter λ corresponds to the lapse rate, reflecting animal failure to respond correctly, which accounts for random errors regardless of the stimulus. The parameter α determines the reflection point of the PF function and indicates the border that divides the adjacent sensory regions. The parameter β is a measure of the sensitivity and determines the slope (*k*) of the curve at the reflection point.

In the TN-task, the stimulus frequency was converted into log-scale in PF fitting. The fitting of the PF was derived using the MATLAB function “*fit*”. The fitting conditions were set by the MATLAB script “*fitoptions*”, in which the search for the optimal fit started with initial parameter conditions at [γ = 0.5, λ = 0.5, α0 = middle point, β = 1], and the search ranges were confined between [0 1] for γ and λ, between [min max] of stimuli quantity for α, [0 100] for β. The inflection point (α) of the fitted curve indicates the perceptual boundary between the Reference and Target categories, and the difference between α and the middle point (α0, the boundaries) can be used to quantity the possible bias (or shift, Δα = α-α0) of the boundary. The sensitivity of the PF at the boundaries could be described by the slope (*k*) at the inflection point derived from the parameter β. The goodness of the fit is quantified by the cost function of normalized root mean square error (*NRMSE*), in which a perfect sigmoid fitting has a value of 1, and a zero or negative value indicates that the fit is no better than a straight line. The PF fitting was performed for each of the behavioral sessions. Following initial behavioral training and reaching behavioral criterion on all tasks, most subsequent behavioral sessions (388/469 = 83%) conducted during neurophysiological recording showed a good sigmoid fit, indicating successful categorical performance.

### Implant surgeries and behavioral methods during neurophysiological recordings

After animals reached behavioral criteria for all trained tasks and attained stable performance, implant surgeries were conducted under aseptic conditions and deep anesthesia with 1–2% isoflurane. A stainless-steel headpost was attached to the ferret skull with bone cement (Palacos MV + Gentamicin, Heraeus). Approximately 2–3 weeks later, following recovery from surgery, animals were gradually habituated to head restraint in a customized stereotaxic frame and retrained on a head-fixed version of the tasks. The stereotaxic frame was positioned on a stable platform in a double-walled soundproof chamber. Acoustic stimuli were presented from a free-field loudspeaker (Manger) located ∼1.2 m directly in front of the animal. During neurophysiological recordings, all sounds were amplified (MA-3, Rane) and presented at 60–75 dB SPL. Animals needed several weeks to retrain and regain successful behavioral performance on the head-fixed version of the tasks.

After the head-restrained animals reattained behavioral criterion performance levels as detailed above, they were prepared for neurophysiological recordings. A small craniotomy (1–2 mm in diameter) was made over brain regions of interest and neurophysiological recordings commenced. Daily recordings were conducted with a 4-tungsten microelectrode array (1–5 MW, FHC) simultaneously inserted into the brain through the craniotomy using an Electrode Positioning Drive (EPS-drive, Alpha-Omega). Raw neural activity traces were digitally acquired/stored by a commercial data acquisition system (Alpha Lab, Alpha-Omega). In each recording session, multiple single units were isolated offline by customized spike-sorting software, based on a PCA and template-matching algorithm (Meska-PCA, NSL). Recordings were made over a period of 12–18 months in each ferret from two brain regions: dorsolateral frontal cortex (FC) and AC. The craniotomies were expanded as needed over the course of the recordings. Only FC data are included in this report.

A typical behavioral neurophysiological session included at least one sequence of three recording epochs in which the animals (1) initially passively listened to a selected stimulus set (from the TN-task or AM-task), (2) then they actively performed the selected task while being presented with the identical stimuli from Epoch 1, and then (3) passively listened once more to the same stimulus set. In each epoch, trials were generated in a pseudorandom order to make sure each of the listed stimuli were equally presented and repeated at least 10 times. Typically, the first behavioral session at each recording site/depth was the TN-task (with all three epochs). If the animal remained motivated to continue behavior and if the units were stable, then recordings continued with a second session with the animals performing the AM-task. Occasionally, the task order was reversed during recordings. Overall, however, there were more TN-task behavioral physiology sessions (*n* = 342) than AM-task (*n* = 127) sessions. In some recording sites (*n* = 119), both TN- and AM-task sessions were made (Table 2).

**Table 2.** Summary of the isolated units and physiological sessions

Paradigm (animals)		Units (Sessions#)			Subtotal
		TN	AM	Both	
	*Gong*	*174* (*43)*	*40* (*9)*	*36* (*9)*	*178* (*43)*
*N*	*Guava*	*368*(*91)*	*241* (*60)*	*186* (*53)*	*423* (*98)*
	**Subtotal**	**542 (134)**	**281 (69)**	**222 (62)**	**601 (141)**
	*Bramble*	*228* (*82)*	*NA*	*NA*	*228* (*82)*
	*Ganges*	*406* (*79)*	*320* (*58)*	*284* (*57)*	*442* (*80)*
*P*	*Nile*	*209*(*47)*	*NA*	*NA*	*209*(*47)*
	**Subtotal**	**843 (208)**	**320 (58)**	**284 (57)**	**879 (209)**
Total		***1,385 (342)***	***601* (*127)***	***506* (*119)***	***1,480* (*350)***

NA, not available. The individual numbers of cells and sessions for each animal are given in italics, whereas the final subtotals are given in Bold.

### Neuroanatomical methods for localization of recording sites

Neurophysiological recordings in the dorsolateral FC were situated 2–8 mm lateral to the midline and 25–30 mm anterior to the occipital crest. This location corresponds to a FC area including both the anterior sigmoid gyrus/premotor cortex (ASG/PMC) and a more anterior dorsolateral FC region of the proreal gyrus (PRG), based on the ferret brain atlas (Radtke-Schuller, 2018). The craniotomy was gradually expanded over the course of 6–18 months of recordings, and in some animals a second separate craniotomy was made over the FC in the opposite hemisphere for additional recordings. Locations of the recording electrodes relative to two landmarks on the surrounding bone cement were noted for later alignment of all electrode penetration sites on the final craniotomy images and a map of all recording sites in each animal.

Following the completion of neurophysiological recordings in each animal, tracer deposits were placed in the FC recording sites to enable subsequent histological confirmation of the recording areas. For each animal, two penetration sites in each identified cortical area were selected. In three of the ferrets (Gong, Ganges, and Nile), iron electrolytic deposits were made at these sites. In the other two ferrets (Guava and Bramble), labeled marking was introduced by injection of a small volume (0.3–0.7 µl) of fluorescent dye [fluoro-emerald (FE) or fluoro-ruby (FR), 0.1 mg/µl in saline; Molecular Probes]. The chosen recording sites for labeling were identified first by responses evoked during task performance. The iron deposits were made by passing a small constant current through stainless-steel electrodes (5–7.5 µA for 300 s). The FE/FR injections were made using a 5.0 µl Hamilton syringe with 30–45° tip. After the electrolytic lesions or fluorescent dye injections, the animals were deeply anesthetized and perfused with a 4% paraformaldehyde solution. In subsequent histology, confirmation of injection sites in 50 µm coronal brain slices of the fixed ferret brains was achieved using a Prussian blue reaction to highlight the iron deposits or by identifying the fluorescent deposits by direct inspection with a fluorescent microscope. Recording sites were successfully identified, confirming localization in dorsolateral FC (including dPFC and rostral part of ASG/PMC) based on the ferret brain atlas (Radtke-Schuller, 2018).

### Methods for neuronal data analysis

Single units isolated from FC recordings were first evaluated by examining responses in three time-epochs relative to stimulus onset: (1) pre-onset baseline (−100 ms pre-onset—i.e., 100 ms before stimulus onset); (2) onset, defined as 0–250 ms following stimulus onset; and (3) sustained, defined as 250 ms poststimulus onset to full stimulus duration. Neuronal activity in each of these three time-epochs was analyzed with respect to sound category (Reference or Target). Neurons that showed significant modulation in at least two of the following six testing pairs were included in the final data analysis: (1) onset response versus baseline response (Reference), (2) onset response versus baseline response (Target), (3) Reference versus Target response (onset), (4) sustained versus baseline response (Reference), (5) sustained versus baseline response (Target), (6) Reference versus Target response (sustained). A total of 1,480 neurons isolated from 350 recording sessions over a period of 12–18 months fulfilled these screening criteria and were therefore included for further analysis (see Table 2 for details). The basic neuronal metrics applied included categorical index (CI), discrimination index (DI), Choice probability (CP), etc. and were described previously (Yin et al., 2020).

#### Clustering neurons based on the mean response profiles

Neuronal activity in FC regions often conveys information about sensory input, stimulus category, attention, decision-making, and motor planning. We sought to analyze these correlations of neuronal activity with internal states and behavior by using principal component analysis (PCA) to extract the major components conveying the dynamics of the response profiles evoked by stimuli. The neurons were then partitioned based on the weights from the dominant components using K-means clustering.

The details of this analysis are discussed and illustrated in Supplementary Figure S1. At first, the response profiles (PSTHs) to Reference or Target sounds categories were averaged from all correct trials and then smoothed with a Gaussian kernel (100 ms in length with 20 ms standard deviation) with a 5 ms moving step. The resulting profiles were then normalized by subtracting baseline activity (obtained by averaging over the 100 ms prestimulus period) and then scaled to between [−1 1] by dividing by the absolute maximum. Neurons with >10 correct trials in both Reference and Target trials were selected to form a 2D data matrix (time bins × neurons). In order to extract the dynamics for overall response profile, the portion of the data matrix from −100 ms prestimulus onset to 1,000 ms poststimulus onset was used to perform PCA decomposition (without centering the columns of data since it had been standardized) in which the rows of the data matrix correspond to observations (time axis) and columns correspond to variables (neurons; Supplementary Fig. S1*A*). The resulting PCs reflect the composition of the dynamic profiles in descending order in terms of the percentage of the total variance explained by the component (Supplementary Fig. S1*B*). The profile contributions in the neuronal response could be estimated by the least-squares solution of a linear equation:

R=Wt*Pr,(2)

where ***R*** denotes the response matrix evoked by Target or Reference (as in Supplementary Fig. S1*A*); the ***Wt*** denotes the profile weights for each neuron; and ***Pr*** denotes the extracted PC profiles which are scaled between [−1 1].

The majority of explained variance (65–84% of total variance across task variants) was captured by the first three PCs, which also exhibited remarkably similar profile patterns across all task variants. We then partitioned the neurons into clusters by using K-means clustering (Matlab function “kmeans” with squared Euclidean distance, 5 time repeats) based on the computed ***Wt****’s* to profiles 1–3 (Supplementary Fig. S1*C*). Three major clusters were found, one with transient responses and two others with sustained average response profiles (Supplementary Fig. S1*D*). This analysis procedure is applied to the data matrix of different stimulus types (the Reference or Target or the combined), of different behavioral contexts (passive or active), and of the neurons in each task (TN or AM) and Paradigms (P, N). Since FC neurons were most strongly driven by active engagement in the task and by the Target sound category, we elected to use only the dataset from Target stimuli to extract the PC profiles and partition the neurons into clusters. The combined dataset (including responses to both Reference and Target stimuli) resulted in comparable PC profiles in which the explained variance by the first three PC profile was slightly lower (by ∼1.8% on average across tasks).

#### Decoding reference and target based on the population neuronal activation patterns

The averaged responses of the neuronal population (e.g., the PSTHs) reveal significant information about the stimuli (Foffani and Moxon, 2004; Bagur et al. 2018). However, the neuronal activity induced by Reference and Target sounds in the same FC population reflected distinct events that played different functional roles during task performance, such as categorizing the stimuli, decision-making/action selection, and execution/maintenance of selected actions. According to the temporal stability hypothesis (Jagadisan and Gandhi, 2022), these multiplexed signals might be aligned with distinct temporal structures of population activity patterns. In this way, the population temporal structure could supplement the rate code (the averaged response) in disentangling the different processes related to the stimuli, decisions, and actions over the time course of behavior. Hence, we adapted the method to decode task stimuli based on the population temporal structures.

Neurons with >12 correct trials for each sound category (both Reference and Target) were included in this analysis. We organized first a 3D pseudo-population data matrix (population trials × neurons × time bins), in which each population trial was constructed by randomly picking a correct Reference or Target trial from each neuron. To remove a potential overtraining effect, the data matrix was limited to include only 12 Reference and 12 Target population trials to make sure that no trial was reselected in each population draw. To assess the temporal structure, the resulting pseudo-population data matrix was then normalized by its Euclidean norm (equivalent to its magnitude) to form a unitary vector in multidimensional neuronal space at each time instant during a population trial (Jagadisan and Gandhi, 2022). Thus, the normalized data matrix reflects a unity length population vector that points in an *n*-dimensional direction at a given time instant in each population trial.

Assuming that the population trials from Reference and Target were aligned with distinct temporal structures associated with different task events, a leave-one-out cross-validation (LOOCV) procedure was used to decode the trial type (Reference or Target) based on the stability of the temporal structure. During the training stage, the template vectors for Reference (***V****ref*) and Target (***V****tar*) were generated by averaging 11 Reference or 11 Target trials at a given time instant, with one Reference/Target trial left out as testing trial. The temporal stability [*S(t, t_0_)*] of the population activation at the time instant (*t*_0_) was then quantified by the dot product of the template vectors (***V****ref* or ***V****tar*) and the single trial test vector [***V****test(t, ref/tar)*] at each time moment of the trial:

$$S(t,t_{0})=Vtemp(t_{0},ref/tar)\cdot Vtest(t,ref/tar).\;(3)$$

A testing trial with a larger stability metric [***S***(*t, t*_0_)] produced from the Target vector (***V****tar*) was then classified as a Target trial or Reference trial. This procedure was repeated 12 times to make each trial serve as a testing trial for a given population data matrix draw. There were a total of 100 population draws in each of the task variants, which yielded a total of 2,400 testing trials (1,200 Reference and 1,200 Target population trials). The temporal stability metric of the testing trials can be visualized in a 2D space constructed by Reference and Target vectors.

#### Extracting β-band rhythms from LFP

LFP waveforms were collected during the same physiological recording sessions as single-unit data. The raw LFP data were first notch filtered to remove 60 Hz noise, then high-pass filtered at 1 Hz (second-order Butterworth) to remove slow drifts, and finally downsampled to 500 Hz. For trials in which electric shock was used during the N-paradigm, the responses around the shock period were set to zero (∼1,200 ms from shock onset). All trial traces were then corrected by the averaged prestimulus level (100 ms before stimulus onset) to further remove the slow drifts on a trial-by-trial basis.

To extract the time–frequency (***TF***) power, the preprocessed LFP data were convolved with a family of complex Morlet's wavelets [***w(t, f_0_)***], which have a Gaussian shape both in the temporal (***σ_t_***) and frequency (***σ_f_***) domain around its central frequency (***f_0_***):

$$w(t,f_{0})\;=\;A\;*\;exp(-t^{2}/2\sigma_{i}^{2})\;*\;exp(2i\pi f_{0}t),\;(4)$$

where ***σ_f_*** ***=*** ***1/(2πσt)*** and a normalization factor ***A*** ***=*** ***(σ_t_ π )^−1/2^*** make total energy equal to 1. A wavelet family is characterized by a constant ratio (***f_0_/σ_f_***). In our analysis, the wavelets we used were defined by ***f_0_/σ_f_*** ***=*** ***5***. The analyzed frequencies ranged from 10 to 115 Hz with 50 equal log spaced bins.

The ***TF*** power of each LFP trial was computed from the squared magnitude of the resulting complex spectrum. The power values were then converted to percent occupancy of the total power for each analyzed frequency channels. The ***TF*** power data were then averaged across trials for different trial types, such as Reference versus Target in terms of stimulus type, or ***Hit***/***Miss*** versus ***Correct reject***/***False alarm*** in terms of task behavior, and standardized by working along time and frequency dimension. The resulting ***TF*** powers were pooled across all recording sites for statistical analyses.

## Results

Five adult female ferrets (1–2 years old) were trained on two different auditory Go-NoGo tasks and opposite behavioral paradigms as discussed later. After initial training, animals were implanted with a headpost, habituated to head fixation, and retrained to behavioral criterion. In subsequent neurophysiological studies, we recorded single-unit activity from FC and AC in all five ferrets. Most of the data from the N-paradigm tasks have previously been analyzed to assess the categorical stimulus representation in primary and secondary AC and FC (Yin et al., 2020). In this study, we report FC data combined from both the N- and P-paradigms, we have but focused here on the role of the FC in executing and controlling the animal actions in the two opposite behavioral paradigms.

### Behavioral paradigms and stimuli

All ferrets were trained on both TN- and/or AM-tasks in which they discriminated two sound categories (Reference versus Target sounds) as detailed in Table 1. Two animals learned to perform the N-paradigm version of the task, while three others performed the P-paradigm version. Thus, there were four task variants in total (two auditory discrimination tasks in each of the N- and P-paradigms). Details of the task structures are depicted in Figure 1*A*, together with the definition of the various trial outcomes, ***Hit***, ***Miss***, Correct Rejection (***CR***), False Alarm (***FA***), and First lick (***FL***) or Last Lick offset (***LL***), and how these outcomes are used to assess animal performance.

All animals successfully learned to perform the tasks (with head restraint in a recording stereotaxic frame), as evidenced by the group averaged lick-rate profiles (Fig. 1*B*) and behavioral performance (Fig. 1*C*) across all recording sessions. Performance of individual animals is provided in Supplementary Figure S2*A*. In the N-paradigm (Fig. 1*Bb1*), animals established a stable lick rate after the water pump was turned on at the beginning of the trial and maintained it during Reference sounds (dark color). Lick rate decreased significantly after onset of the “danger” Target sounds (light color). In the P-paradigm (Fig. 1*Bb2*), animals refrained from licking at the beginning of a trial and exhibited a significantly higher lick rate after presentation of “safe” Target sounds than to References. As seen in Figure 1*C*, significant performance was achieved in all four groups of paradigms and tasks, as assessed by *d′* (derived from ***Hit*** and ***FA*** rates). However, it is also evident that the type of performance errors reflected strongly the urge of the animals to drink in the context of the two paradigms. Thus, most errors in the N-paradigm were ***Misses*** (Fig. 1*Cc1*) arising from animals failing to stop licking during the shock window occurring after a Target sound. In contrast, in the P-paradigm (Fig. 1*Cc2*), most errors were ***FAs*** as the animals failed to withhold licking after Reference sounds. To further characterize behavioral performance, we computed PFs by fitting the likelihood of behavioral response in relation to the stimulus metric with a 4-parameter logistic function (see Materials and Methods, Behavioral assessment and psychometric functions fitting). Using these logistic functions, we could estimate the likelihood of a response error attributed to pure guessing [guess rate (l) estimation] or to inattention or motor errors [lapse rate (g) estimation]. The animals likely developed distinct behavioral strategies in the two paradigms to maximize their water gain and minimize negative reward (shock or timeout). For example, as shown in Figure 1*D*, N-paradigm animals (*d1*, *d2*) tended to downward-shift their **PF**s, choosing a conservative strategy or disengagement, while P-paradigm animals (*d3*, *d4*) tended to upward-shift the PFs (more evident in the AM-task and difficult portions of the TN-task; Supplementary Fig. S2*B*), choosing a liberal strategy or improved vigilance (see details in Supplementary Fig. S2*C*). The different behavioral strategies were also evident in the different single-unit responses driven by the error trials from the two paradigms (Supplementary Fig. S3) and also by the dynamics of the choice probability which confirmed a significant behavioral modulation of the single-unit activity only during the P-paradigm as we shall illustrate and discuss later.

### Response types across different behavioral conditions and stimuli

**Figure 2.**
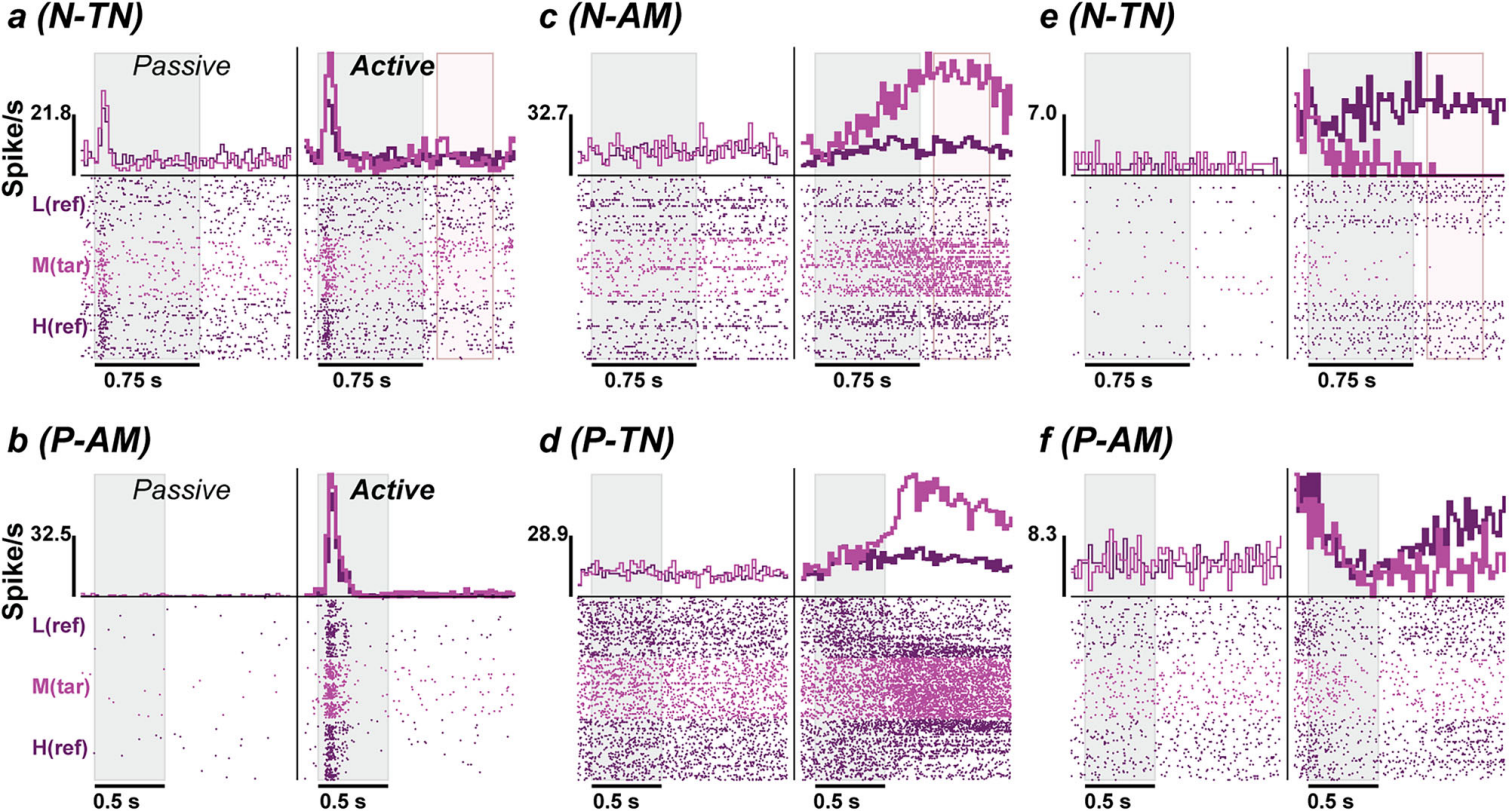
Diversity of single-unit responses in FC during performance of different tasks. Six representative examples of single-unit neuronal responses are shown, spanning both stimulus types (**TN**, **AM** noise) and the two reward N- and P-paradigms (**N**, **P**). In each of the six panels, the **Target** (**Reference**) responses in pink (purple) are shown in the passive (left-half of each panel) and active (right-half of panels) states. Overall, responses to **Targets** were stronger than to **References** and greater in the active than in passive states. Five of the cells (***b–f***) exhibited behaviorally gated responses, being responsive only in the active task condition. Two cells (***a***, ***b***) exhibited phasic responses to tones (***a***) or to AM noises (***b***). Both responded more vigorously to **Target** than **Reference** and more strongly during the active than passive states. Two cells (***c***, ***d***) showed gradual buildup of a sustained excitatory response to AM (***c***) and Tone (***d***) stimuli. A different pattern was seen in cells (***e***, ***f***) that exhibited an increase in baseline firing rate during active task engagement while responding to task stimuli with sustained suppression both to tones (***e***) and AM noise (***f***) stimuli. The suppression was deeper during **Targets** than to **References** and in the active compared with passive states.

We recorded from 1,480 neurons in total, with 601 neurons in the two N-paradigm animals, and 879 neurons in the three P-paradigm animals (Table 2). The majority of recorded units were confirmed to be anatomically located in the dorsolateral FC (dlFC) regions ranging from the dorsal prefrontal cortex (dPFC) to the rostral part of anterior sigmoid gyrus/premotor cortex (ASG/PMC; Supplementary Fig. S4*A*) based on the ferret brain atlas (Radtke-Schuller, 2018). Single units displayed a substantial diversity of responses to Target and Reference stimuli in all behavioral tasks as illustrated in Figure 2 where single-unit responses are depicted both as spike-rasters and poststimulus time histograms (PSTHs). Responses exhibited a significant enhancement in the active (task-engaged) condition, compared with the quiescent passive listening condition, regardless of task reward paradigm or stimulus type (TN or AM). The enhanced response in the active state was most apparent in the Target responses but was also seen more modestly in the responses to Reference stimuli (Fig. 2*b*; Supplementary Fig. S4*B,a–d*,*g*,*i*). While some neurons showed weak transient (Fig. 2*a*; Supplementary Fig. S4*B,g*) or sustained responses during the passive state (Supplementary Fig. S4*B,a*), the activity of many neurons was behaviorally gated—with virtually no responses to either Reference or Target stimuli in the passive condition, becoming vigorous only during task engagement (Fig. 2*b–f*,*h*,*i*). These findings are consistent with earlier results from ferret FC (Fritz et al., 2010; Yin et al., 2020).

Response patterns varied widely across the neuronal population, ranging over three major types: transient (or phasic; Fig. 2, left panels), sustained excitatory (Fig. 2, middle panels), sustained suppressed responses (Fig. 2, right panels) with some neurons displaying hybrid responses (examples in Supplementary Fig. S4*B,b,h,i*). However, for any given neuron, its response pattern remained generally the same in all conditions apart from displaying modulation in strength depending upon the behavioral context (passive/active) or stimulus type (Target/Reference). For example, the two cells shown in Figure 2*a*,*b* were from different animals and reward paradigms, yet they exhibited similar phasic responses in both passive and active conditions, to Reference and Target, and to TN and AM stimuli. The two other common response patterns—sustained excitatory and sustained suppressed responses shown in Figure 2,*c–f*—were most strongly responsive to Target stimuli in the active condition. An interesting feature observed in many cells' responses (Fig. 2*e*,*f*) was a significant increase in baseline responses during the active (relative to passive) state. A possible interpretation of the changes of baseline activity during the active state is discussed and illustrated later. We shall provide below a detailed analysis of these three basic response types and their potential functional significance across the entire FC neuronal population.

### Averaged population PSTHs exhibited similar temporal profiles across tasks

**Figure 3.**
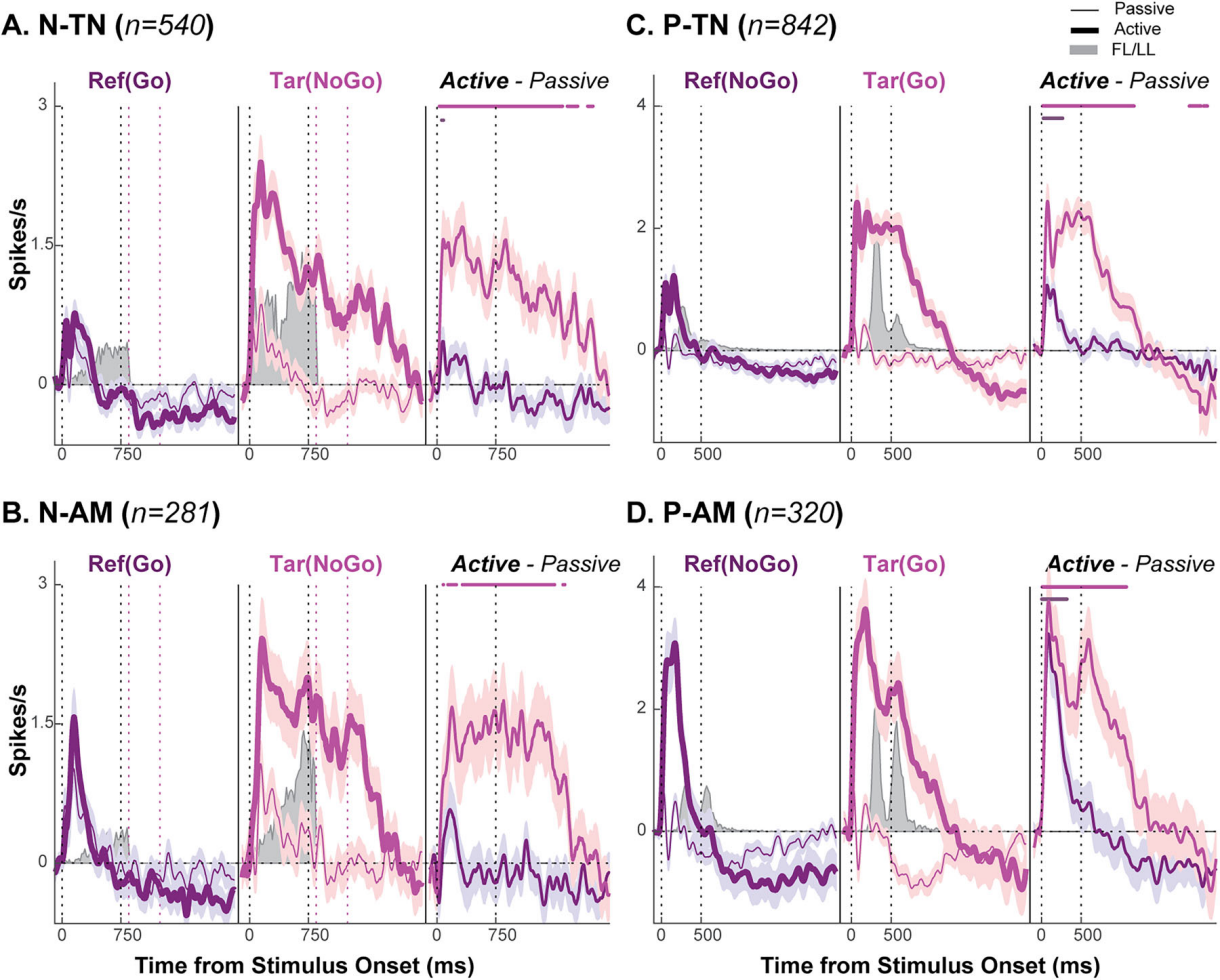
Distinct population response profiles evoked by Reference (Ref) and Target (Tar) stimuli in different task paradigms (N, P) and stimuli (TN, AM). The four panels are constructed from all FC neurons recorded in the N-paradigms (***A***, ***B***) and P-paradigm (***C***, ***D***) and both TN (***A***, ***C***) and AM (***B***, ***D***) stimulus types. The population PSTHs to Reference (purple line, left subpanel) and Target (pink line, middle subpanel) sound categories were corrected by the prestimulus baseline firing rate (measured in the 100 ms window prior to stimulus onset) in each experimental epoch. The PSTHs with thin lines indicate the response during the passive listening state, and the bold lines show responses during the active state (task engagement). The right subpanels of the plots depict the net changes in **Target** and **Reference** responses between the active and the passive states (i.e., Active–Passive). The shaded regions around each line are the standard errors at each moment. All PSTHs were smoothed with a 100 ms Gaussian kernel (with *20* ms standard deviation) sliding at 5 ms steps. The lines at the top of each right subpanel indicate the significance of the changes (by *t* test, *p* < 0.05 with Bonferroni’s correction for repeated measures). The filled gray areas in each of the left and center subpanels display the distributions of the behavioral reaction times to Reference (***FA*** trials) or Target (***Hit*** trials) sounds [measured by first lick (***FL***) time after stimulus onset in the P-paradigm or the last lick stop (***LL***) time in the N-paradigm; Fig. 1*A*]. The vertical black dotted lines indicate stimulus onset and offset time, and the shock window for the N-paradigm is indicated by the vertical magenta dotted lines.

FC neurons exhibited similar response dynamics for encoding of categorical information in the two paradigms and tasks (Supplementary Fig. S5). To gain a broad view of the overall FC response patterns, the population PSTHs were assembled from isolated single units in each of task variants (Fig. 3), grouped by Target (pink) and Reference (purple) sound categories. Three features of FC activity emerged when viewing the population PTSH responses in the various conditions in Figure 3: (1) responses to all task stimuli in the passive state (thin lines) were relatively weak and predominantly transient; (2) during the active state (thick lines), Target responses (pink) became strongly enhanced and more sustained in character; while (3) Reference responses (purple) remained far more phasic than Target responses. Reference responses were also significantly weaker than Target responses in all conditions, except for the P-AM task (Fig. 3*D*) where they were comparable. The enhancement of responses in the active state is highlighted by displaying the contrast (or difference) between the Active and Passive states, shown in the third column of all panels in Figure 3. An additional feature also emerged, namely, (4) a slightly delayed suppressive response to both Reference and Target stimuli, a response feature that (as we discuss later) may potentially play an important functional role in the behavioral responses of the animals.

Despite the overall similarity in the population responses across tasks and paradigms, there were a few features that distinguished the population PSTH responses between the various tasks which may be attributed to the details of the behavior or the diverse stimuli (Fig. 3). For instance, one difference between the N- and P-paradigms (left vs right panels) was the longer sustained Target responses observed in the N-paradigms (∼2 vs 1 s), a difference that reflects specific differences between the durations of behavioral engagement (Fig. 1*A*) following the Target stimuli in the two paradigms (i.e., in the N-paradigm the ferrets have to stop licking and to maintain this behavior beyond the shock window, whereas in the P-paradigm, the ferrets could initiate licking at any time within the response window). Another difference concerns the AM versus TN categorization tasks (bottom vs top panels). In the active state, onset responses to AM Reference stimuli were stronger than Reference responses to the TN stimuli in both the N- and P-paradigms, becoming comparable to (but still somewhat smaller than) the phasic portion of the Target response. One possible reason for the relatively strong Reference and Target phasic responses in the AM-task is that it is more difficult, possibly requiring more attention and longer reaction times during task performance, factors that are potentially reflected in the phasic portions of the response (Yin et al., 2020).

To summarize, Target responses in both reward paradigms exhibited stronger and more sustained responses compared with the phasic Reference responses. This similarity of FC responses across the two opposite paradigms is striking given the marked differences between the associated behavioral actions and strategies (Fig. 1; Supplementary Fig. S2). Furthermore, since these similar FC responses preceded diverse behavioral reactions in the different task variants [as reflected by the overlaid distributions (in gray) of reaction times to Target (***Hits***) or Reference (***FA***) sounds in Fig. 3], it suggests that the FC response does not precisely encode the motor details of the actual actions of licking or holding but rather it likely encodes a more abstract representation that broadly controls and gates these opposite actions as we discuss below.

### Principle components of the PSTH responses: three cell clusters

**Figure 4.**
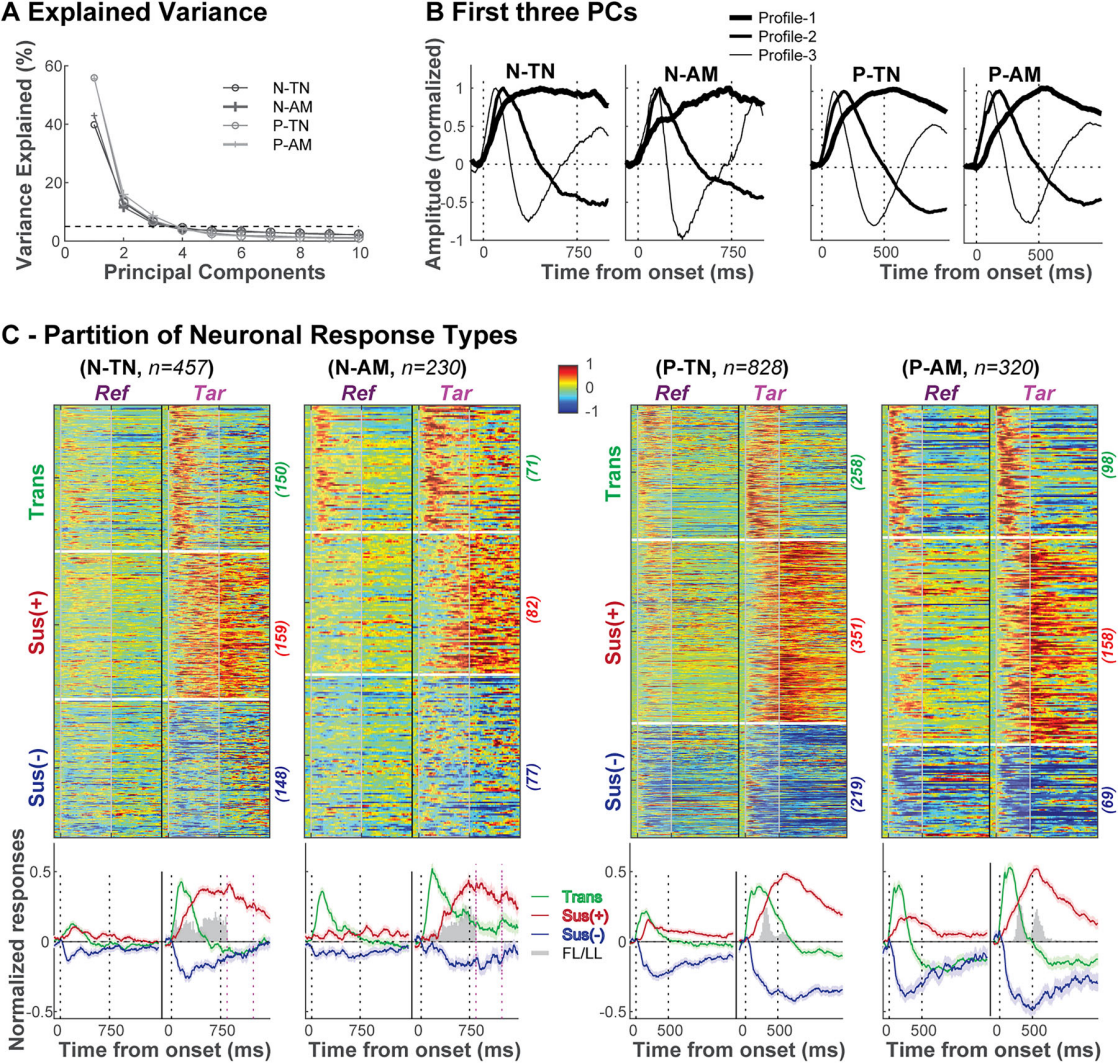
Principle component analysis and clustering of the FC neuronal responses. ***A***, PC analysis of responses in each of the four different task variants of Figure 3 yields three major components, where each accounts for over 5% of the variance (horizontal dashed line) and together account for an average of ∼75% (67.3, 68.2, 78.5, and 83.6%) of the total profile variance among the analyzed time segments. ***B***, The three largest PCs of each task variant are similar across all task variants. ***C***, Clustering all cell responses. Computing the projection weights associated with the three PCs in each task yields three similar basic types of neuronal responses. Details of the clustering are given in the Materials and Methods and Supplementary Figure S1. Each of the four panels shows the raster responses to the Reference (left) and Target (right) stimuli and are organized into three response types (clusters): Transient (***Trans***, Top); Sustained excitatory [***Sus(+)***, middle]; Sustained inhibitory [***Sus(−)***, bottom]. The overall average Reference and Target PSTH responses from each of the three cell clusters within a task are shown in the bottom row of panels. They are colored in green, red, and blue corresponding to the ***Trans***, ***Sus(+)***, and ***Sus(−)*** clusters, respectively. The filled gray areas display the distributions of behavioral reaction times to Target (***Hit*** trials) for the N-paradigm (***LL***) and for P-paradigm (***FL***) as in Figure 1*A*.

As described earlier in Figure 2, individual neurons exhibited a wide variety of response dynamics, especially to Target sounds during active task performance. Therefore, to further explore the nature of these response profiles, we performed Principal Component Analysis (PCA) over the data matrices formed by Time (the averaged PSTHs evoked by correct Reference/Target trials) × neurons (as detailed in the Materials and Methods and Supplementary Fig. S1*A,B*). In total, data from four task variants [N- and P-paradigms × 2 stimuli (TN & AM)] were used; the top 3 principle components (PCs) contributed significantly to explaining the total variance across the neuronal population (Fig. 4*A*, >5% dashed line) and exhibited comparable response profiles (Fig. 4*B*), in which the combination accounted for 67–84% of the total explained variance. We projected onto these three PCs all cell responses during the active episodes, and then using K-means, we clustered the resulting coefficients into three groups (as detailed in the Materials and Methods and Supplementary Fig. S1*C,D*). The heatmaps of the raster responses sorted by cell clusters are shown in Figure 4*C* (top row of panels), segregated among the four task variants. The population PSTHs corresponding to each cluster are plotted in Figure 4*C* (bottom row of panels). These population partitions reveal three basic response patterns that were remarkably consistent across all tasks: (1) a first cluster of neurons was mostly weighted by profile-2 and/or profile-3 (Fig. 4*B*, Supplementary Fig. S6*A*), which displayed a rapid transient response pattern depicted by the green waveforms (***Trans***); (2) a second cluster was dominated by profile-1 with positive weights (Supplementary Fig. S6*A*). It exhibited a longer latency and a sustained excitatory response as shown by the red waveforms [***Sus(+)***]; (3) a third cluster was also dominated by the extracted profile-1 but with negative weights (Supplementary Fig. S6*A*) and had a medium latency sustained inhibitory response as depicted by the blue curves [***Sus(−)***]. These three clusters emerged in both Target and Reference responses, varying only by their relative strength. In all cases, Target responses were significantly larger, and the combined three PC profiles gave rise to all the PSTH dynamics described earlier in Figure 3. The earliest transient response (***Trans*** Cluster) reflects the sensory and categorical information about the task stimuli (Yin et al., 2020), combined with information on the decision process, behavioral choice, and transformations to the selected action. The transient response is followed closely by a buildup of sustained responses both from the excitatory ***Sus(+)*** and inhibitory ***Sus(−)*** clusters which continue throughout the behavioral response of the animal to the Target stimulus. This sustained bidirectional (positive/negative) modulation of the Target response may reflect the inhibition of the ongoing status quo behavior, and the initiation and maintenance of the selected action during the trial, which is consistent with earlier findings [Fritz et al. (2010), their Fig. 3].

Interestingly, neurons in both the Trans and Sus(−) clusters showed a tendency to increase their baseline firing rates (measured during a 100 ms prestimulus silence period) as the animal switched into the active task context from passive listening (Supplementary Fig. S6*B*). We reemphasize that the composition and dynamics of all task Reference and Target responses were similar, differing primarily by the relatively stronger Target responses in each of the three Clusters.

### Decoding reference and target stimuli based on population activity patterns

**Figure 5.**
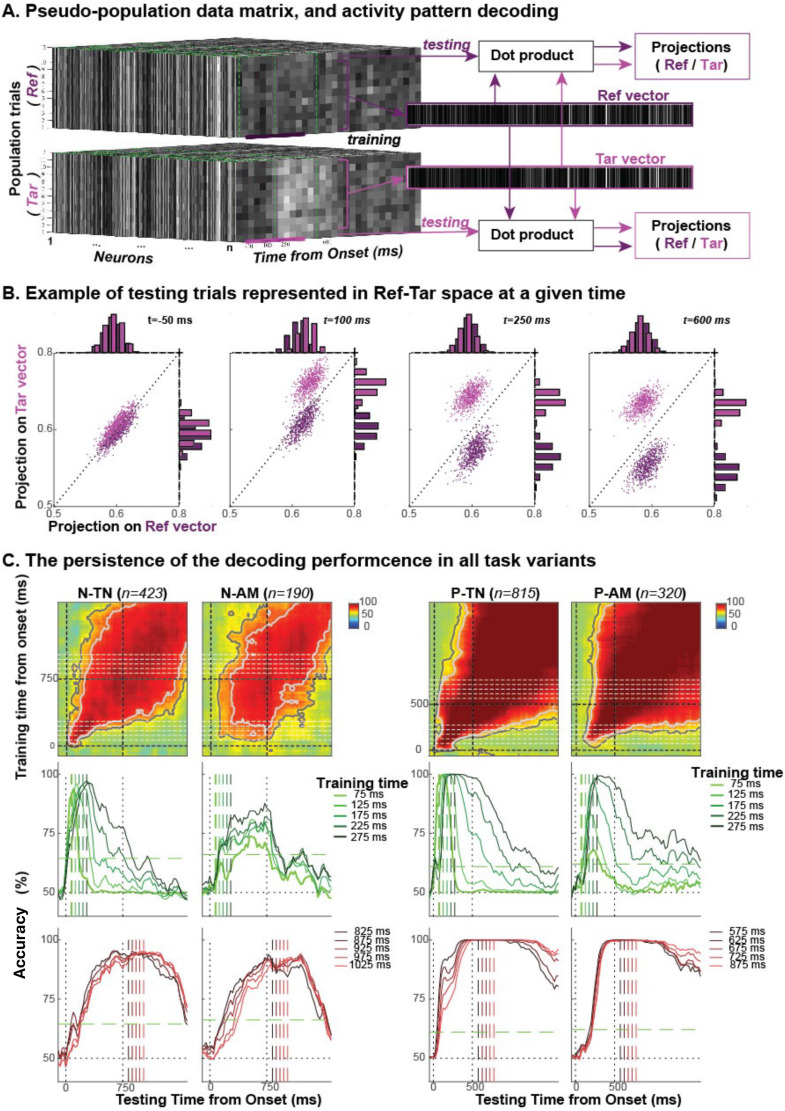
Decoding Reference and Target stimuli from population responses. ***A***, The flowchart shows the procedures for training the Reference and Target vectors to decode the stimulus categories (e.g., the Reference or Target) from the response dynamics of the pseudo-population trials. Details are provided in the text and in the Materials and Methods. ***B***, Scatterplot shows the projections of the responses from the testing population trials onto the trained Target and Reference decoder vectors (*y-* and *x*-axis, respectively). They are computed at different times throughout the population trials as indicated in the different panels. The projections from Reference (purple) and Target (pink) population trials are indistinguishable when the decoder was trained before stimulus onset (e.g., the leftmost panel was trained at −50 ms, and the pink/purple projections are mixed). However, they become segregated (decodable) when trained after stimulus onset, as early as 100 ms from stimulus onset and maintained through the trials. ***C***, Decoding dynamics are similar across task variants. Each of the four columns of panels display the persistence of the decoder performance across the entire trial in decoding the Reference/Target stimuli, when the decoder was trained at different times relative to the stimulus onset. The heatmaps (the top row of panels) show the changes in accuracy of a given decoder trained at a given time (depicted along the *y*-axis) in decoding Reference and Target stimuli across the testing times (depicted along the *x*-axis) and reveal similar patterns across all N-paradigm (left) and P-paradigm (right) tasks. During the early period, the decoders change rapidly within tens of milliseconds (i.e., the accuracy does not persist for very long over time) as reflected by the narrow cross-sectional patterns in the middle row of panels (75–275 ms from stimulus onset, extracted along the dashed white horizontal lines on the heatmaps). The heatmaps exhibit significantly wider red regions at later times, indicating that the decoders persist for hundreds of milliseconds as seen in the bottom row of panels which display the cross sections of the heatmaps at late training times (825–1,025 ms from stimulus onset, as indicated by the dashed white horizontal lines on the heatmaps). Further detailed interpretations of all these plots are available in the text and in the Materials and Methods.

FC population activity integrated diverse neural signals that likely played different functional roles during task performance, such as categorizing the stimuli (Reference vs Target), decision-making (Go vs NoGo), and execution/maintenance of selected actions (licking vs restraining licking). These multiplexed neural signals may have distinct temporal structures that can be disentangled and observed over the time course of the behavior (Jagadisan and Gandhi, 2022). To explore these dynamics in the Target and Reference population responses, we trained a decoder to discriminate between the two stimulus categories (e.g., Reference vs Target) based on the temporal structure of the neuron population as demonstrated in flowchart of Figure 5*A* (details in Materials and Methods). At first a pseudo-population 3D data matrix was constructed from segregated responses of the Reference and Target stimuli. Each data matrix included 24 population trials (12 Reference and 12 Target), with one randomly selected correct trial from each neuron. To minimize overtraining effects, trials from each neuron were only selected once in each population draw. The resulting pseudo-population data matrix was then normalized by its Euclidean norm to form a unitary vector in multidimensional neuronal space at each time instant during a trial.

The flowchart extending to the right in Figure 5*A* illustrates the details of the decoding method with the population activation pattern using the “leave-one-out” cross-validation procedure (see Materials and Methods). For each testing trial, the projections on the “Target-Vector” were compared with the “Reference-Vector” which reflected the trial and thus each testing trial could be classified as Reference or Target.

An example of the results from this approach is shown with data from the P-paradigm, with a total of 1,200 (100 draws) population trials for each stimulus type (Fig. 5*B*). The scatterplots show the representation of those trials in population neuronal space constructed by Target-Vector (*y*-axis) versus Reference-Vectors (*x*-axis) at different points in time from stimulus onset (−50, 100, 250, and 600 ms) and illustrate the gradual change in those Target (purple) and Reference (pink) population trials represented in the constructed neuronal space. The representation dynamically changes from an indivisible mixture (−50 ms before stimulus onset) to become reliably separate clusters within 100 ms after stimulus onset. This is quantitatively confirmed by the bar plots along the two axes representing the distributions of the projections.

More insights into the decoding performance can be gleaned from the results shown in Figure 5*C*, where the topmost row of heatmaps illustrate decoding performance in each of the four task variants with the color map indicating decoder training and testing accuracy at each time point from stimulus onset (chance performance at 50% level is colored green). Overlaid contour lines indicate one or two standard deviations above chance (>50% level is colored in progressively darker shades of red). The panels in the middle and bottom rows of Figure 5*C* illustrate cross sections of the testing performance heatmaps (Fig. 5*C*, top panels) at various training times. For instance, training early in the trial (from 75 to 275 ms after sound onset, as depicted by the dashed vertical lines) already reveals above-chance discrimination between Target and Reference stimuli in all tasks. But the trained decoders from this early period only persist for tens of milliseconds. In contrast, when training the decoder later in the trial (175–275 ms after sound offset) when the animal was already engaged in its contingent behavior (Fig. 5*C*, bottom panels), reliable discrimination emerged from as early as ∼300 ms after stimulus onset, and the decoder remained stable throughout the rest of the behavior for hundreds of milliseconds.

These findings therefore suggest that the categorical decisions based on the sensory input that distinguishes Reference from Target stimuli likely occur relatively early (within 100 ms) following stimulus onset (Fig. 5*C*, middle row panels) and that they are mediated by the earliest transient (phasic) responses (Fig. 4, Cluster 1 cells ) that are similarly evoked early after stimulus onset. This is consistent with previous studies (Yin et al., 2020; Supplementary Fig. S5). In contrast, the behavioral actions associated with the two stimulus categories became stably encoded later in the trial (Fig. 5*C*), suggesting that the combination of sustained responses of Cluster 2 and 3 populations were responsible for encoding actions elicited by the Reference and Target stimuli. This evolution of information in the population responses during the trial, from encoding stimulus category to encoding behavioral action, was also observed in the FC β-band responses that are discussed next.

### The β-band response patterns in the LFPs of the FC

**Figure 6.**
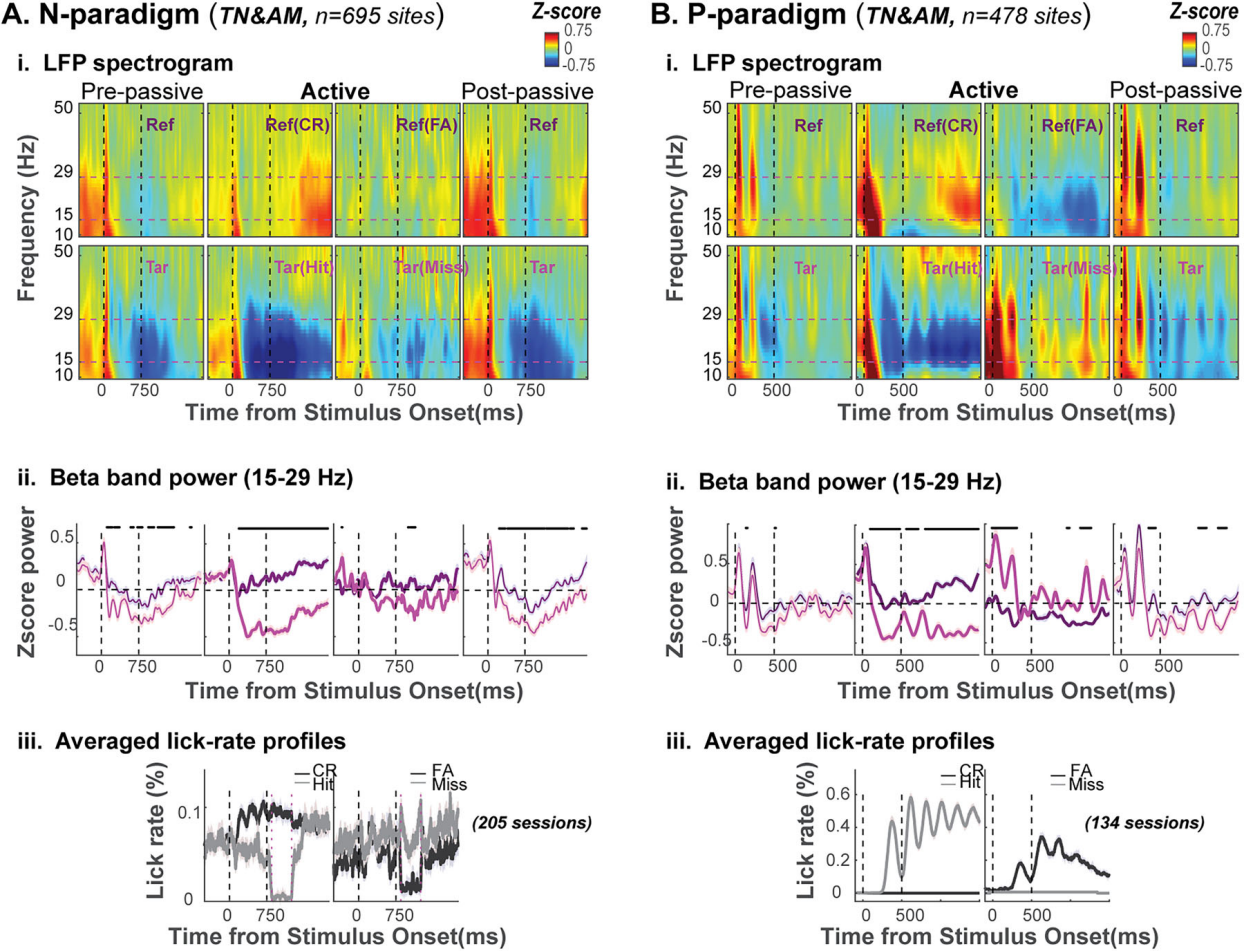
β-band (15–29 Hz) rhythms derived from LFP's of FC responses. ***A***, β-band responses during N-paradigms. ***i***, Each panel depicts the wavelet transforms of the LFPs around the range of the β-band marked by the horizontal dashed lines (magenta). Reference (**Ref**) responses are shown in the top row of panels from left to right during pre-passive, active task engagement, and post-passive. The second row of panels depicts the same information but for Target stimuli. The correct trials [Correct Reject (***CR***) and ***Hits***] and incorrect trials (***Miss*** and ***FA***) are displayed in separate panels during active task engagement (two middle panels). ***ii***, This row of line plots contrasts the dynamics of the integrated β-band power from **Reference** and **Target** sounds. The black dots above the lines indicate a statistical significance of the contrast at the corresponding time moment between the induced β-band power of the two stimulus types (by paired *t* test, *p* < 0.05 with Bonferroni’s correction for repeated measures). ***iii***, Panels depict the lick-rate profiles for correct response (***Hit*** and ***CR***, left panel) and error response (***Miss*** and ***FA***, right panel) trials during active task engagement. ***B***, β-band responses during P-paradigms. The information depicted in all panels is analogous to that of the ***A*** panels. The vertical dashed lines depict stimulus onset and offset in all panels.

One of the key objectives of this study is to explore how FC responses encode diverse actions driven by the same sounds in different behavioral contexts. Specifically, how are the opposite contingencies in response to Target sounds (i.e., start-licking in P-paradigms vs stop licking in N-paradigm) reflected in the Target responses? Previous studies of FC functional responses have demonstrated diverse rhythmic responses as LFP signatures that correlate with behavioral states, e.g., Δ-band (2–3.5 Hz) in consolidating memories (Harmony, 2013), θ-band (3.5–8 Hz) in working memory (Han et al., 2026), α-band (8–14 Hz) in attentional focus (Athanasiou et al., 2018), and γ-band (30–64 Hz) in learning and information processing (Bastos et al., 2020; Ichim et al., 2024). FC rhythmic responses in the β-band (15–29 Hz) have been shown to strongly correlate with sensorimotor and motor actions such as stopping and switching behaviors (Diesburg et al., 2021; Tatz et al., 2023; Horst et al., 2024), as well as the control of goal-directed processing of sensory information and the timing of motor output (Lundqvist et al., 2024; Antzoulatos et al., 2016). To explore the dynamics and response strengths of these rhythms, we derived the integrated response power from the LFPs recorded simultaneously with the FC single-unit data in the context of our two paradigms. Figure 6 displays details of the analysis methods and highlights of the results focused near the β-band range (15–29 Hz), and findings from the expanded frequency range (2–64 Hz) are highlighted in Supplementary Figure S8.

Figure 6 illustrates the grand average of the induced power spectrogram from wavelet transforms of the LFPs evoked by Reference (top-half) and Target (bottom-half) stimuli across all recording sites from the N-paradigm (Fig. 6*A*) and the P-paradigm (Fig. 6*B*). The data from the TN and AM-tasks for each paradigm are combined (details of the wavelet transform computations and normalization procedure are available in the Materials and Methods). The power spectrograms for each task variant are provided in Supplementary Figure S7, which shows a similar picture between TN- and AM-tasks within each of the two behavioral paradigms. In both figures, panels *i* are organized from left to right to depict responses in pre-passive (i.e., before task engagement), active (i.e., task performance), and post-passive behavioral states. The integrated power of the β-band responses (15—29 Hz) is displayed below in panels *ii* for both Target (pink) and Reference (purple) responses in each state.

The β-band responses differ considerably from other LFP frequency band responses in that they remain similar across the two behavioral (N- and P-) paradigms, during both passive and active epochs as illustrated in Figure 6*i*,*ii*. This similarity of β-band responses across the two opposite behavioral paradigms is consistent with the results from the single-unit PSTH responses (Fig. 3). For instance, the most prominent response dynamic in both paradigms occurs during the active Target responses (Fig. 6, middle panels of *i*,*ii*), where there was a brief, transient onset response to the stimulus followed by a deep depression of the β-band power that lasted throughout the behavioral response, usually recovering gradually back to baseline toward the end of the trial. Similar but much weaker dynamics were observed in the Reference responses, where the post-onset depression was shallower and recovered more rapidly. In the passive state (left panels of *i*,*ii*), both Reference and Target response dynamics remained the same, albeit in a weaker form. The response was seen most clearly in the post-task passive state where the memory of the active responses was clearly persistent. The β-band power depression during the active and post-passive responses are also consistent with earlier single-unit results from PFC [Fritz et al. (2010), their Fig. 6].

When the animals committed behavioral errors during task performance such as ***FA*** (to References) or ***Miss*** (to Targets), the β-band responses displayed different patterns as seen in the two middle panels (top and bottom) of Figure 6*i*,*ii*. Broadly speaking, error responses in both the N- and P-paradigms are consistent with the animals' cognitive state and their actions, i.e., Reference ***FA***s resemble Target ***Hit***s, while Target ***Miss***es resemble Reference ***CR***s. Consequently, and as expected, the β-band power in Reference FA responses is more suppressed than ***CR***s, whereas Target ***Miss*** responses become less suppressed than ***Hit***s. The amount of response modulations due to performance errors are far more salient in the P- than N-paradigms, a difference that likely reflects the divergent behavioral strategies in the two paradigms as discussed and illustrated previously in Figure 1 and Supplementary Figures S2, S3.

Finally, to emphasize the substantial dissociation between the **FC** β-band dynamics and licking, we show in Figure 6*iii* the lick-rate profiles during Target and Reference trials for both correct (left panels) and erroneous (right panels) trials in the N- and P-paradigms, respectively. Consider for example the elevated lick rates following the Target in the P-paradigm (Fig. 6*Biii*, ***Hit***-trace in the left panel). In contrast, the lick rate plunged to zero during the shock period of ***Hit*** trials in the N-paradigm (Fig. 6*Aiii*, ***Hit***-trace in the left panel; 850–1,250 ms interval between the two red dotted lines). Nevertheless, despite these opposite lick rates in the two paradigms, the β-band suppression following the correct Target (***Hit***) behavior (Fig. 6*i*,*ii*, two middle panels) was similar in both the N- and P-paradigms. The same contrast between lick rates and β-band responses applies to the correct Reference (***CR***) behavior, where the absent versus sustained licking (Fig. 6*iii*, ***CR***-traces in the left panels) corresponds to a similar shallow depression in the Reference ***CR*** responses during the active state (Fig. 6*i*,*ii*).

## Discussion

### Summary of the experimental results

This study explored how behavior and its associated cognitive functions are encoded in ferret FC as they categorized sounds in the context of Go-NoGo tasks (Yin et al., 2016, 2020). Ferrets learned to perform the tasks in one of two paradigms designed so that while the acoustic stimuli and categories were identical, the paradigms were opposite in their reward valence and task structure. Animals learned to categorize both AM and TN stimuli, although displaying distinct behavioral strategies in the P- and N-paradigms (Fig. 1, Supplementary Fig. S2).

Although there was diversity of single-unit responses during all stages of the tasks (Fig. 2, Supplementary Fig. S4), recordings nevertheless could be summarized succinctly by the average population PSTHs of Figure 3, where substantially similar response features were observed across all tasks despite the opposite contingencies, actions, and behavioral strategies associated with the stimuli. Two shared features were that all PSTHs exhibited larger responses during active than passive states and to Target than to Reference stimuli (Figs. 2, 3).

The PCA dimensionality reduction of all Target and Reference responses yielded three dominant PCs that were similar across all task variants (Fig. 4*A*,*B*) and explained up to 80% of total response variance (Fig. 4*A*). The population averaged responses could be shown to be composed of different weightings of these three PC profiles (Fig. 4*C*,*D*). Based on these weights, the recorded FC neurons could be clustered into three subgroups: ***Trans***, ***Sus(+)***, and ***Sus(*−*)*** (Fig. 4*C*). As a population, the early transient responses provided a reliable decoding of Target versus Reference stimuli beginning within 100 ms after stimulus onset (Fig. 5*B*,*C*). This gave way to a sustained and stable response over the subsequent hundreds of milliseconds (Fig. 5*C*). We conjecture that the initial response phase in FC is related to stimulus categorization (Supplementary Fig. S6; Yin et al., 2020). For Targets, we propose that this is immediately followed by a “decision” leading the animal to stop ongoing action and switch to a different behavior. This represents an abstract, higher-level representation of stimulus-induced change in behavior (Koechlin et al., 2003; Gruber and McDonald, 2012; Baladron and Hamker, 2020, 2024; Vaidya and Badre, 2022).

The β-band responses (extracted from the LFPs) were also uniform across all tasks, with substantially greater response modulations to Targets than to References and during active than passive states (Fig. 6*A*,*B*). Active Target β-band responses consisted of a transient “positive” onset followed by a sustained depression. In contrast to the uniformity of the β-band responses, the rhythmic power of the Δ-, θ-, α-, and γ-bands was quite variable, thus diverging from the uniformity of the cells' spiking responses in the P- and N-paradigms, suggesting that these rhythms may instead reflect differences in reward valence or other task variables rather than action control.

Modulations of the β-band power in the FC and STN have previously been reported as neurophysiological signatures of pausing (initial enhanced burst), followed by stopping or switching behavior (β-depression) in tasks with Go-NoGo or Pause-and-Switch behavior (Engel and Fries, 2010; Meyer and Bucci, 2016; Wessel and Anderson, 2024), which are tasks associated with voluntary switching from ongoing movements (Horst et al., 2024), exactly what our animals do in response to Target stimuli.

### Functional relevance of target and reference responses

**Figure 7.**
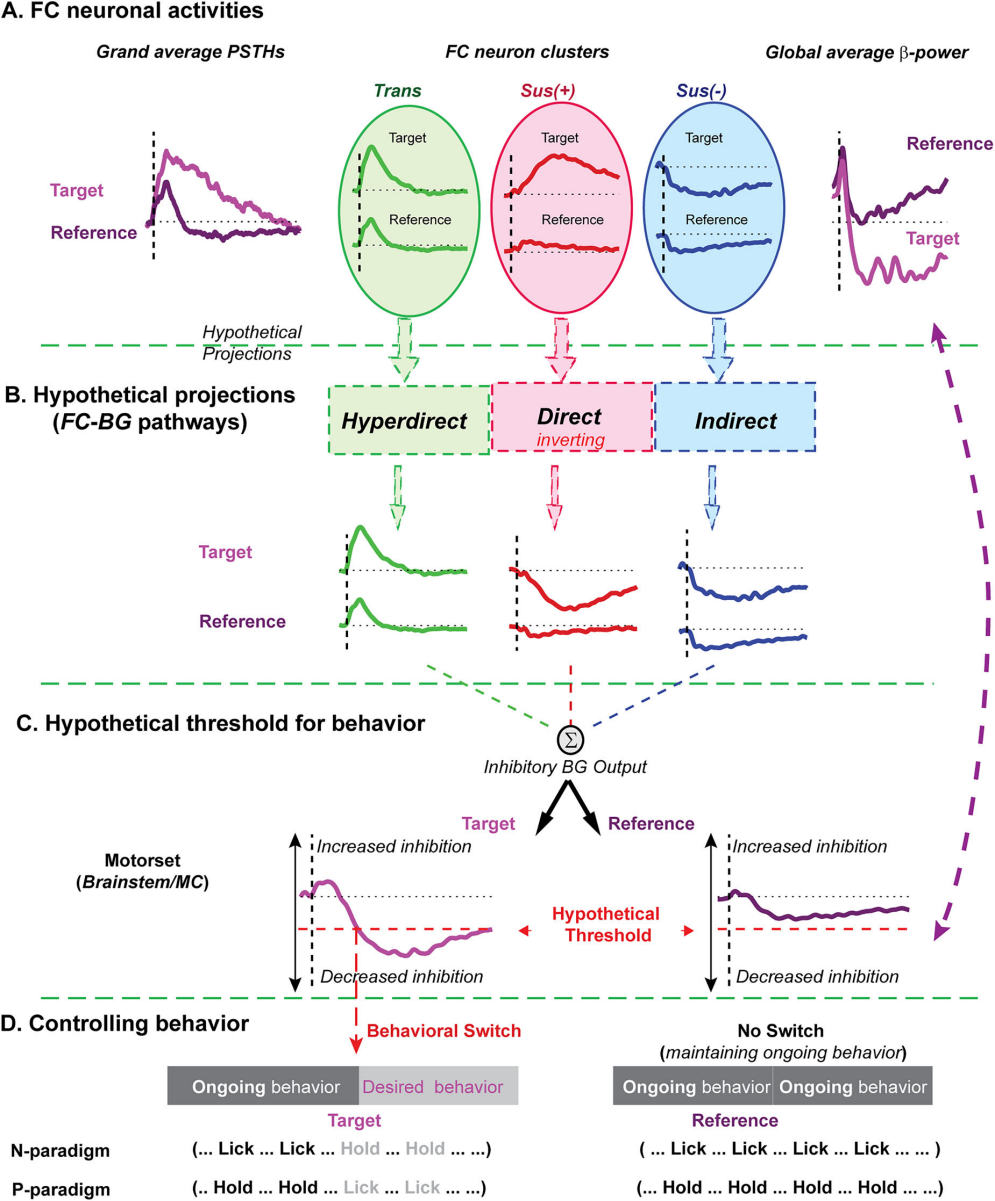
Proposed model of functional cortico-striatal projections from the three FC cell clusters. ***A***, Grand average FC neuronal responses: The responses are averaged across all tasks and sources to generate grand average PSTHS according to stimulus types, Target (pink) or Reference (purple). From left to right, the response PSTHs are from all neurons, the three neuron clusters [***Trans***, ***Sus(+)**,* and ***Sus(*−*)***], and the *β-band* power. ***B***, Hypothetical projections (FC–BG pathways): It is conjectured that the three distinct FC cell clusters may project in parallel to the striatum and then through the nuclei of the three BG pathways: Hyperdirect, Direct, and Indirect. Note that the FC responses from the ***Sus(+)*** cluster become inverted. ***C***, Hypothetical threshold of the motor-set: The integrated BG output is approximately represented by the sum of the three FC cluster responses propagating through the BG pathways, after which it may activate a motor-set through the thalamocortical loops. The integrated output responses are larger to Targets (left) than to References (right), causing stronger disinhibition of the motor-set. Such output waveforms closely resemble the grand average β-band power responses depicted in the rightmost panel of ***A*** (as symbolized by the dashed double arrow). ***D***, Schematic of the proposed functional role of Target and Reference stimuli on task behavioral actions and performance in the N- and P-paradigms. When the integrated BG output activity induced by the Target (but not the Reference) stimuli exceeds a hypothetical threshold (the horizontal dashed and arrow lines depicted in ***C***), we conjecture that this leads to behavioral switching from ongoing (status quo) behavior to task desired behavior by disinhibition of the downstream motor-related regions.

Planned movements activate extensive brain networks including FC, motor cortices, and cortico-striatal-thalamo-cortical pathways (Kravitz et al., 2010; Sippy et al., 2015). Although FC is known to play a key role in planning actions, stopping an ongoing action, or rapidly switching to a more optimal action, we wondered what specific role the FC responses may have played in shaping the ferret behavior in our study. Given the diversity of stimuli and behaviors across both paradigms, the uniformity of their associated responses in the FC is extraordinary and likely reflects a common functional thread of events with a shared meaning. To provide greater insight into the FC representation, we computed in Figure 7*A* the grand average response PSTHs of all neuron population (leftmost panel), of the three neuron clusters [***Trans***, ***Sus(*****−*)***, and ***Sus(+)***; middle panels], and of the β-band power during the active states (rightmost panel). As expected, all closely recapitulate earlier response features, observed at the single-unit, population, and LFP levels.

We next considered the possible common functional role that Target and Reference stimuli have in all tasks, particularly in the opposite contexts of the N- and P-paradigms. Previous studies have emphasized that FC encodes abstract, higher-level rules (Koechlin et al., 2003; Gruber and McDonald, 2012; Vaidya and Badre, 2022). If the FC responses are interpreted as acting at a higher level of abstraction, then Target stimuli could serve to signal to the animal the need to switch (change) its ongoing behavior to an appropriate alternative. In contrast, Reference stimuli signal to the animal to maintain its current behavior. Therefore, in this view, Target responses in FC do not reflect details of the different motor-sets (licking vs nonlicking) but rather mediate a behavioral change (switching actions), while Reference responses promote maintenance of the status quo and continuation of ongoing action (Engel and Fries, 2010). Our results show that Target sounds produce similarly sustained responses (at a single-cell, population level, and in the LFP β-band) whether the animal suddenly withholds licking (N-paradigm) or initiates licking (P-paradigm; Figs. 3, 6). This abstraction of task structure is highlighted in the schematic of Figure 7*D* which translates the N- and P-paradigms to this shared and more abstract description (Koechlin et al., 2003; Vaidya and Badre, 2022).

Given the close interactions between the FC and BG, one challenge in integrating our findings with the existing BG functional literature is that in most Go-NoGo and Stop signal studies (Wessel and Anderson, 2024), there has been a literal interpretation of “Go” and “NoGo” choices as “start-action” versus “stop-action.” A central insight from our results is that FC responses and by inference BG outputs do not necessarily reflect movement per se, but rather the abstract control of desired actions that may or may not include movement. Thus, withholding licking in the N-paradigm tasks (actively switching from licking to nonlicking) is abstractly an action like any other, despite the apparent behavioral inaction; after all, even during the nonlicking state, there is an active component of tongue retraction.

### Possible contributions of the three FC clusters to the FC–striatal projections

How could the three basic clusters of FC responses contribute to task behavior? It is known that FC projections through the basal ganglia (BG) are essential for movement control and play a key role in action selection, initiation, and modulation of motor activity. According to the classical BG motor model, two pathways—Direct (inverting) and Indirect (Fig. 7*B*)—exert opposing control over motor behavior, whereas a third rapid Hyperdirect pathway issues an excitatory output to initiate pauses. It is classically postulated that intended “Go” movements can be promoted via the Direct pathway, whereas the Indirect pathway mediates suppression of competing motor programs and conveys stop signals by a “No-Go” response (Frank, 2005; Bariselli et al., 2019).

Our findings of three basic clusters serving opposite motor actions suggest a different hypothetical model. We speculate that the interplay between these FC responses propagating through the three major cortico-BG projections underlies how the FC reshapes and maintains actions in response to sensory stimuli. Specifically, the FC clusters may initiate and reflect the loop activity arising from the three cortico-BG pathways. We speculate that the ***Trans***, ***Sus(+)***, and ***Sus(*−*)*** clusters dominate the Hyperdirect, Direct, and Indirect pathways, respectively (Fig. 7*B*). When converging to the BG's output nuclei, the Direct pathway inverts its FC ***Sus(+)*** inputs, whereas the Indirect pathway preserves the polarity of FC ***Sus(*−*)*** inputs. The third (Hyperdirect) ***Trans*** FC projection is a rapid excitatory monosynaptic input to the subthalamic nucleus (STN) and then to the BG output (Fig. 7*B*). The proposed FC cluster activities converge on BG's output nuclei, exhibiting a remarkably similar profile (Fig. 7*B*) to the global average β-band power profiles (Fig. 7*A*, rightmost panel).

The rationale for this hypothesis, aligning the output of the three functional FC clusters we have observed, with the three major cortico-striatal pathways, is the observed dynamic sequence of FC response features in our study that is broadly consistent with experimental evidence. Initially, in the quiescent passive listening state, FC responses to any acoustic stimuli are minimal, and hence there is a weak FC–BG drive. Consequently, we conjecture that the BG outputs send a high level inhibition to the motor-set through the related motor regions (brainstem and/or motor cortex through the motor thalamus) that maintain the current motor state of the animal. To initiate a different voluntary movement, we hypothesize that the FC–BG pathways must reduce the BG inhibitory output significantly, nominally below a threshold level (marked by the dashed red line in Fig. 7*C*), to enable the motor-set to initiate a new, appropriate motor action. During (active) task engagement, when strong FC responses to Target stimuli cause BG outputs to decrease below threshold and release BG inhibition of the motor-set (Fig. 7*C*, left panel), it becomes possible for the animal to switch or change a previous (ongoing status quo) action and initiate a different action (Fig. 7*D*). In contrast, since the FC responses to Reference stimuli are relatively weak, we conjecture that they would not reach threshold at the BG output (Fig. 7*C*, right panel), thus maintaining the ongoing status quo, by continued inhibition of the motor-set.

### Future experimental validations

Although the hypothetical tripartite projections of different FC clusters to the BG (Fig. 7) and the specificity of the cortico-striatal-thalamo-cortical loop projections are all highly speculative conjectures (Pasquereau and Turner, 2023; Baladron and Hamker, 2024), they are consistent with many aspects of the current literature and likely underlie analogous tasks where a different action is initiated by a change from the status quo upon correctly perceiving a “Switch Cue.”

We have not demonstrated here that the same FC clusters are involved during the P- and N-paradigms, since our recordings occurred in animals trained on only one paradigm. Since there is some evidence that appetitive and aversive reward may recruit distinct FC populations (Ye et al., 2016), it is crucial to test our model in animals trained on both paradigms and can switch between them. Other important tests are needed in future experiments, e.g., to assess the generality of our FC responses in other species (mice, primates, and humans) and to explore whether FC responses in different paradigms involving action switching (e.g., 2AFC) would exhibit similar response regardless of actions (e.g., pulling levers or making saccades). We further predict that simultaneously recorded STN and FC responses should be correlated during switching behavior (at Target presentation) for ***Sus(*−*)*** and ***Trans*** neurons, but not for ***Sus(+)*** neurons. We also predict that selective optogenetic inactivation of FC Sus(+) or Sus(−) clusters would differentially impact the ferret's switching behavior.

### Conclusions

Our study suggests that FC responses to a Target stimulus encode and control subsequent voluntary actions in a sequence of three abstract states that are underlined by the three response clusters: (1) ***Trans*** is induced by a sensory stimulus categorized as Target (triggers a new desired action) or Reference (remain in status quo behavior); (2) ***Sus(*−*)*** initiates a Switch from ongoing behavior to the new action; and (3) ***Sus(+)*** sustains the newly initiated action. The details of how this FC transformation of sensory stimuli to consequent actions comes about and especially the role of the BG, thalamus, and various motor and other sensory cortical regions remain to be explored and clarified in future studies.
